# Complete Genome Sequence of Microbacterium foliorum NRRL B-24224, a Host for Bacteriophage Discovery

**DOI:** 10.1128/MRA.01467-18

**Published:** 2019-01-31

**Authors:** Daniel A. Russell, Rebecca A. Garlena, Graham F. Hatfull

**Affiliations:** a Department of Biological Sciences, University of Pittsburgh, Pittsburgh, Pennsylvania, USA; University of Delaware

## Abstract

We report the complete annotated genome sequence of Microbacterium foliorum NRRL B-24224, a type strain isolated from the phyllosphere of grasses and a commonly used host for bacteriophage discovery. The genome contains no identifiable prophage or CRISPR or restriction-modification system, which suggests that it may continue to be a fruitful host for phage discovery.

## ANNOUNCEMENT

*Microbacteria* are Gram-positive rod-shaped aerobic bacteria in the order *Actinomycetales* which have been associated with growth promotion and drought resistance in plants (1–3), spoilage in packaged meats (4), and even presence on cell phone screens (5). Microbacterium foliorum NRRL B-24224 (acquired by NRRL from DSM [DSM 12966]) is a type strain originally isolated from the phyllosphere of grasses and is available from the NRRL culture collection (6). Its relative ease of growth, short doubling time, and natural habitat have made it a productive host for use with the phage discovery protocols of the SEA-PHAGES program (7; https://seaphages.org/sections/2018/Fall/). To date, more than 680 phages have been isolated on NRRL B-24224, of which 98 have been sequenced and divided into 9 clusters and 4 singletons (8).

A single colony of NRRL B-24224 was picked and grown to saturation in a peptone yeast calcium (PYCa) medium. Genomic DNA was isolated by lysing the cultures of NRRL B-24224 in a 3110BX Mini-BeadBeater for 45 s, treating them with RNase, and performing a phenol-chloroform extraction. A barcoded library for Illumina sequencing was prepared with an NEB Ultra II FS kit and run on an Illumina MiSeq system, which produced just over 3 million 150-base single-end reads. From the same DNA sample, a second library for Oxford Nanopore sequencing was prepared with a rapid barcoding kit (SQK-RBK004) and run on a MinION sequencer with a FLO-MIN6 (R9.4) flow cell for 6 h, which produced ∼30,000 reads with an average length of ∼5.7 kb. These 2 sets of reads were assembled with Unicycler v0.4.4 (9), with the -s flag for the Illumina reads and the -l flag for the Nanopore reads and default settings. Unicycler was used to assemble the reads into a single circular bacterial contig, with 126-fold Illumina coverage and 47-fold Nanopore coverage, which was evaluated and adjusted for accuracy and completeness using Consed v29 and custom scripts as previously described (10).

The complete genome of NRRL B-24224 is 3,568,138 bp long and circular, and it has a G+C content of 68.7%. No plasmids or extrachromosomal elements were identified. The first base and orientation were selected to correspond to previously sequenced microbacterial genomes, with *dnaA* as the first gene in the genome. Its closest sequenced relative is Microbacterium foliorum strain 122, with a whole-genome NCBI BLASTn query coverage of 78% and a maximum identity of 88%.

The NRRL B-24224 genome sequence was submitted to GenBank and annotated with NCBI’s Prokaryotic Genome Annotation Pipeline (11), which identified 3,264 protein-coding genes, 45 tRNA genes, and 2 rRNA operons. A search with PHASTER (12) revealed no intact prophages, such that neither prophage-encoded superinfection immunity nor prophage-mediated heterotypic defense systems should constrain the types of phages isolated on this strain (13). The genome does contain an intact type II VapBC toxin-antitoxin system but no apparent restriction-modification or CRISPR system. These findings suggest that NRRL B-24224 has many useful attributes as a host for exploring the diversity and evolution of the bacteriophage population.

### Data availability.

The complete genomic sequence for Microbacterium foliorum NRRL B-24224 is available at GenBank under accession number CP031425. Sequencing reads are available at the Sequence Read Archive under accession numbers SRX4863569 (Nanopore) and SRX4863570 (Illumina).
